# The effects of *Crocus sativus* (saffron) and its constituents on nervous system: A review

**Published:** 2015

**Authors:** Mohammad Reza Khazdair, Mohammad Hossein Boskabady, Mahmoud Hosseini, Ramin Rezaee, Aristidis M. Tsatsakis

**Affiliations:** 1 *Neurogenic Inflammation Research Center and Department of Physiology, School of Medicine, Mashhad University of Medical Sciences, Mashhad, Postal Code 9177948564, Iran*; 2 *Student Research Committee, Mashhad University of Medical Sciences, Mashhad, Iran*; 3 *Neurocognitive Research Center and Department of Physiology, School of Medicine, Mashhad University of Medical Sciences, Mashhad, Postal Code 9177948564, Iran*; 4 *Department of Physiology and Pharmacology, School of Medicine, North Khorasan University of Medical Sciences, Bojnurd, Iran*; 5 *Center of Toxicology Science and Research, Division of Morphology, Medical School, University of Crete, Heraklion, Crete, Greece*

**Keywords:** *Crocus sativus*, *Nervous system*, *Safranal*, *Crocin*, *Saffron*

## Abstract

Saffron or* Crocus sativus* L. (*C. sativus*) has been widely used as a medicinal plant to promote human health, especially in Asia. The main components of saffron are crocin, picrocrocin and safranal. The median lethal doses (LD50) of *C. sativus* are 200 mg/ml and 20.7 g/kg *in vitro *and in animal studies, respectively. Saffron has been suggested to be effective in the treatment of a wide range of disorders including coronary artery diseases, hypertension, stomach disorders, dysmenorrhea and learning and memory impairments. In addition, different studies have indicated that saffron has anti-inflammatory, anti-atherosclerotic, antigenotoxic and cytotoxic activities. Antitussive effects of stigmas and petals of *C. sativus* and its components, safranal and crocin have also been demonstrated. The anticonvulsant and anti-Alzheimer properties of saffron extract were shown in human and animal studies. The efﬁcacy of *C. sativus* in the treatment of mild to moderate depression was also reported in clinical trial. Administration of* C. sativus* and its constituents increased glutamate and dopamine levels in the brain in a dose-dependent manner. It also interacts with the opioid system to reduce withdrawal syndrome. Therefore, in the present article, the effects of *C. sativus* and its constituents on the nervous system and the possible underlying mechanisms are reviewed. Our literature review showed that *C. sativus* and its components can be considered as promising agents in the treatment of nervous system disorders.

## Introduction

*Crocus sativus* L (*C. sativus*), commonly known as saffron, is a small perennial plant belonging to the family of Iridaceas. This plant is cultivated in many countries including Iran, Afghanistan, Turkey and Spain (Abdullaev, 1993 ▶). The stigmas of *C. sativus* are known to contain carotenoids, α-crocetin and glycoside crocin (responsible for saffron yellow color) and picrocrocin, the aglyconesafranal (responsible for saffron aroma) (Fernández and Pandalai, 2004 ▶; Champalab et al., 2011), the antioxidant carotenoids lycopene and zeaxanthin and vitamin B2(Vijaya Bhargava, 2011).

It has been shown that *C. sativus* stigma aqueous extract and its constituents, crocin but not safranal enhanced the sexual activity in male rats (Hosseinzadeh et al., 2008 ▶). Saffron and its constituentscrocin and safranal are also shown to be potent oxygen radical scavengers (Assimopoulou et al., 2005 ▶; Mashmoul et al., 2013 ▶; Farahmand et al., 2013).

In traditional medicine, *C. sativus* has been frequently used as an herbal sedative, antispasmodic, aphrodisiac, diaphoretic, expectorant, stimulant, stomachic, anticatarrhal, eupeptic, gingival sedative and emmenagogue (Nemati et al., 2008 ▶).* C. sativus* was experimentally shown to be effective in relieving symptoms of premenstrual syndrome (PMS). Following administration of saffron, a signiﬁcant effect was observed in cycles 3 and 4 in the Total Premenstrual Daily Symptoms and Hamilton Depression Rating Scale which indicates the efﬁcacy of *C. sativus* in the treatment of PMS (Agha-Hosseini et al., 2008 ▶). 

Aqueous (500 mg/kg) and ethanolic extracts of *C. sativus* petals reduced blood pressure in a dose-dependent manner in rats (Fatehi et al., 2003 ▶). Administration of the aqueous extract of saffron petals (500 mg/kg) reduced blood pressure from 133.5±3.9 to 117±2.1 mmHg in rats. This reduction was postulated to be due to the effect of the extracts on the heart itself, total peripheral resistance or both (Fatehi et al., 2003 ▶). In rats isolated vas deferens, contractile responses to electrical field stimulation (EFS) were decreased by the petals extracts (Fatehi et al., 2003 ▶). EFS-induced contractions of vas deferens were shown to be mediated by noradrenaline and adenosine triphosphate (ATP) released as co-transmitters from sympathetic nerves (Hoyle and Burnstock, 1991 ▶). The ethanolic extract made more pronounced changes in EFS in rats isolated vas deferens whereas in guinea pig ileum, the aqueous extract of the plant was more effective (Fatehi et al., 2003 ▶). Crocin analogs isolated from saffron remarkably increased the blood flow in the retina and choroid and facilitated retinal function recovery; therefore, it could be used to treat ischemic retinopathy and/or age-related macular degeneration (Xuan, 1999 ▶). One study suggested that saffron exerted a signiﬁcant cardioprotective effect by preserving hemodynamics and left ventricular functions (Sachdeva et al., 2012 ▶). Administration of *C. sativus* extractinpatients who had normal white blood cells (WBC) count, significantly increased WBC compared to crocin or placebo. Moreover, other hematologic factors were not changed significantly during 3 months of the study (Mousavi et al., 2015 ▶). 

A potent stimulatory effect of *C. sativus* extract and safranal on β2-adrenoreceptors has also been reported (Nemati et al., 2008 ▶; Boskabady et al., 2010 ▶). In addition, blocking effect of safranal on muscarinic receptors (Boskabady et al., 2010 ▶) and the inhibitory effect of *C. sativus* on histamine (H1) receptors was reported, which proposed a competitive antagonistic effect for *C. sativus* on histamine (H1) receptors (Boskabady et al., 2010 ▶).

An in vitro study showed the inhibitory activity of saffron and crocin on amyloid beta-peptide ﬁbrillogenesis and its protective action against H_2_O_2_–induced toxicity in human neuroblastoma cells (Papandreou et al., 2006 ▶, 2011). Additionally, administration of saffron (60 mg/kg body weight, i.p.) to normal and aged mice for one week, significantly improved learning and memory (Papandreou et al., 2011 ▶). Also, *in vitro* studies have confirmed the neuroprotective effects of saffron and its constituents in amnesic and ischemic rat models (Hosseinzadeh and Sadeghnia, 2005 ▶; Ochiai et al., 2007 ▶).

Considering clinical and animal experimental studies, the present review explores the important effects of* C. sativus* and its constituents on nervous system*.*

## Methods

Information of this review article was collected by searching for the key-words "*Crocus sativus"*, "nervous system", "clinical application", "animal studies", "crocin", "crocetin" and "safranal" in databases namely ISI Web of Knowledge, Medline/ Pubmed, Science direct, Scopus, Google Scholar, Embase, Biological Abstracts and Chemical Abstracts. 

***C. sativus*** **constituents**

More than 150 compounds have been identified in saffron stigma including colored carotenoids (e.g. crocetin and crocins as glycosidic derivatives), colorless monoterpene aldehydes, volatile agents (e.g. safranal and picrocrocin which are the bitter components), etc. (Bathaie and Mousavi, 2010 ▶). The traces of non-glycosylated carotenoids unrelated to crocetin are β-carotene, lycopene and zea-xanthin (Ríos et al., 1996 ▶). Ethanolic extract of saffron has visible absorption peaks at 427 and 452 nm. When excited at 435 nm, saffron emits at 543 nm (Horobin and Kiernan, 2002 ▶). 

Crocetin isolated from saffron is one of the two principal chemicals responsible for the red color of saffron (Martin et al., 2002 ▶). Crocetin constitutes approximately 0.3% of the total weight of the saffron stigma (Escribano et al., 1996 ▶, Dris and Jain 2004 ▶). Crocetin can function as an acid (anionic) dye for biological staining because it has a carboxyl group at each end of the polyene chain which is easily dissolved in aqueous alkali solutions at pH ≥ 9. Crocetin is mostly present as trans isomer but cis-crocetin and its glycosides are also present in saffron as minor components (Melnyk et al., 2010 ▶).

Crocin belongs to a group of natural carotenoid commercially obtained from the dried stigma of *C. sativus*. It has a deep red color, forms crystals with a melting point of 186 ^o^C and is easily soluble in water. Crocin is responsible for the color of saffron. Structure of crocin was elucidated by Karreeet al (1935) ▶. It is the main pigment of saffron (approx. 80% of pigment content). Pure crocin can be isolated from saffron extract and is directly crystallized (Karrer et al., 1932 ▶). Crocin is not orally absorbed. Crocins are hydrolyzed to crocetin before or during intestinal absorption, and the absorbed crocetin is partly metabolized to mono and diglucuronide conjugates (Asai et al., 2005 ▶). 

Crocins, accounting for almost 6–16% of saffron dry weight (Gregory et al., 2005 ▶), are hydrophilic chemicals. α –crocin (crocin 1) is a carotenoid which comprises the majority of crocins found in saffron. It could be so easily dissolved in water that is used as color additive (Melnyk et al., 2010 ▶). The other color compounds of saffron are carotenoids and glycosidic, alpha-carotene, beta-carotene, lycopene, Zeaxanthingentiobioside, glycoside, gentio-glycoside, beta-crocetin di-glycoside and gama-crocetin.

Safranal (which is fat soluble) and pigments of the crocetin carotenoid are bitter, but the most important cause of saffron bitterness is picrocrocin (Abdullaev, 1993 ▶). Saffron lipophilic carotenoids are lycopene, alpha- and beta-carotene and zeaxanthin(Winterhalter and Straubinger, 2000 ▶; Tarantilis and Polissiou, 1997 ▶). Kaempferol has also been found in alcoholic extract of saffron petals (Gregory et al.,2005 ▶). Flavonoids especially lycopene, amino acids, proteins, starch, resins and other compounds have also been shown to be present in saffron (Assimopoulou et al.,2005 ▶). Saffron also has trace amounts of thiamine and riboflavin (Alonso et al.,2001 ▶).

**Anticonvulsant effects**

In Iranian folk medicine, *C. sativus* had been used as an anticonvulsant herb (Khosravan, 2002 ▶). Experimental studies also confirmed saffron anticonvulsant effects in rats and mice (Sunanda et al., 2014 ▶; Khosravan, 2002 ▶). Saffron at the doses of 400 and 800 mg/kg showed a significant antiepileptic activity in pentylenetetrazole (PTZ)-induced seizure model in a dose-dependent manner. However, saffron at the dose of 200 mg/kg did not significantly suppress PTZ- induced seizures (Sunanda et al., 2014 ▶). The anticonvulsant activities of aqueous and ethanolic extracts of saffron have been demonstrated in mice using maximal electroshock seizure (MES) and PTZ models (Khosravan, 2002 ▶).

Safranal (0.15 and 0.35 ml/kg, i.p.), reduced PTZ-induced seizure duration, delayed the onset of tonic convulsions and protected mice from death but crocin (200 mg/kg, i.p.) did not show anticonvulsant activity (Hosseinzadeh and Talebzadeh, 2005 ▶). Intraperitoneal administration of safranal (72.75, 145.5 and 291 mg/kg) decreased the frequency of minimal clonic seizures (MCS) and generalized tonic clonic seizures (GTCS) (Hosseinzadeh and Sadeghnia, 2007 ▶). Safranal also attenuated the acute experimental absence seizures which was attributed to modifications of benzodiazepine binding sites of GABA_A_ receptor complex (Sadeghnia et al., 2008 ▶).

**Anti-Alzheimer effects**

*Basic studies*

Alzheimer's disease (AD) is described pathologically as deposition of amyloid β-peptide (Aβ) fibrils. The aqueous-ethanolic (50:50, v/v) extract of *C. sativus* stigmas has good antioxidant properties -higher than those of carrot and tomato- in a concentration and time-dependent manner which was accompanied by inhibition of Aβ fibrillogenesis. The trans-crocin-4, the digentibiosyl ester of crocetin was the main carotenoid constituent which inhibited Aβ fibrillogenesis (Papandreou et al., 2006 ▶). Intracerebroventricular (ICV) injection of streptozotocin (STZ) to rodents has been frequently used as an animal model for sporadic AD (Lannert and Hoyer, 1998 ▶; Labak et al., 2010 ▶; Veerendra Kumar and Gupta, 2003 ▶). It has been previously revealed that treatment by *C. sativus*extract (30 mg/kg) for 3 weeks could significantly improve cognition deficits induced by ICV injection of STZ in rats (Khalili et al., 2010 ▶).Crocin (30 mg/kg) has also been shown to have an antagonizing effect on the STZ-induced cognitive deﬁcits in rats (Khalili and Hamzeh, 2010 ▶).

Geromichaloset al. (2012) ▶ showed that the saffron extract had a moderate (up to 30 %) inhibitory activity on acetyl-cholinesterase (AChE) and inhibited acetylcholine breakdown which is the main therapeutic approach for AD (Geromichalos et al., 2012 ▶).

*Clinical studies*

Administration of saffron 30 mg/day (15 mg twice daily) was found to be as effective as donepezil for treatment of mild-to-moderate AD in the subjects of 55 years and older (Akhondzadeh et al., 2010a ▶ ). In addition, the frequency of saffron extract side effects was similar to those of donepezil except for vomiting, which occurred more frequently in the donepezil group (Akhondzadeh et al., 2010a ▶). In another study, 46 patients with mild-to-moderate AD were treated by saffron for 16 weeks. The results showed that the cognitive functions in saffron-treated group were signiﬁcantly better than placebo (Akhondzadeh et al. 2010b ▶).

**Antidepressant and anti-** **schizophrenia** **effects**

*Basic studies*

Crocin and ethanolic extracts of saffron are known to have antidepressant effect in rodents. Using forced swimming test, it was shown that crocin (50–600 mg/kg) reduced immobility time while increased climbing time (Hosseinzadeh et al., 2003). In other studies,effectiveness of antidepressant activity of *C. sativus* extract was described (Karimi et al., 2001 ▶; Yang Wang et al., 2010). The petroleum ether and dichloromethane fractions were suggested to be the active parts of corms of *C. sativus*. The petroleum ether fraction of the extract of *C. sativus* L. corms mainly contained n-tridecane, n-tetradecane, n-pentadecane, diethyltoluamide, n-catane and n-heptadecane, etc. (Yang Wang et al., 2010). 

Kaempferol, a *C. sativus* petal constituent also reduced immobility behaviors in mice (100 and 200 mg/kg) and rats (50 mg/kg) (Hosseinzadeh et al., 2007 ▶). A decreased time of immobility in rodents caused by selective serotonin re-uptake inhibitors such as fluoxetine may explain the antidepressant effects of the plant (Cryan and Lucki, 2000 ▶; Lucki, 1997 ▶). The antidepressant effect of aqueous and ethanolic extracts of *C. sativus* petal and stigma has been shown in mice (Karimi et al., 2001 ▶). Major constituents of saffron, safranal and crocin, also had antidepressant activity in mice (Hosseinzadeh et al., 2004 ▶). 

The effectiveness of *C. sativus* as a treatment for depression in animal model was shown in Table 1. 

*Clinical studies*

In a randomized and double-blind clinical trial study, saffron supplementation statistically improved the mood of subjects compared to the placebo group. For six weeks, 30 mg/day of saffron was given and subjects were evaluated based on the Hamilton Depression Rating Scale (HAM-D) (Akhondzadeh et al., 2005 ▶). Another similar study by Noorbala et al. (2005) ▶ revealed that six-week administration of saffron extract (30 mg/day) was effective in the treatment of mild to moderate depression. These effects were similar to the effects of ﬂuoxetine (Noorbala et al., 2005 ▶) and imipramine 100 mg/day (Akhondzadeh et al., 2004 ▶). Therapeutic benefits of petals of *C. sativus* in the treatment of mild to moderate depression have also been suggested (AkhondzadehBasti et al., 2007 ▶). The efficacy of co-administration of hydro-alcoholic extract of *C. sativus* (40 or 80 mg) and fluoxetine (30 mg/ day) was also investigated in a double- blind randomized clinical trial for six weeks. The results revealed that a dose of *C. sativus* 80 mg plus fluoxetine was more effective than that of *C. sativus* 40 mg and fluoxetine to treat mild to moderate depressive disorders (Moosavi et al., 2014 ▶). 

**Table1:** The effectiveness of *C. sativus* as a treatment for depression in animal models

**Constituent**	**Animal**	**Doses**	**Results**	**References**
**Aqueous and ethanolic extract **	Mice	(0.2–0.8 g/kg)	The aqueous and ethanolic extracts of stigma, reduced immobility time.	Hosseinzadeh et al., 2003
**Aqueous and ethanolic extract , Crocin**	Mice	(50–600 mg/kg)	Reduced immobility time and increased swimming time.	Hosseinzadeh et al., 2003
**Aqueous and ethanolic extract ,Safranal**	Mice	(0.15–0.5 mL/kg)	Reduced immobility time and increased swimming time.	Hosseinzadeh et al., 2003
**Kaempferol**	Mice	100 and 200 mg/kg	Reduced immobility behaviors	Hosseinzadeh et al., 2007

Short-term administration of saffron (30 mg/day) capsules for six weeks was also shown to be as effective as ﬂuoxetine (40 mg/day) in improving depression symptoms in patients who were suffering from major depressive disorder (MDD) after undergoing a percutaneous coronary intervention (Shahmansouri et al., 2014 ▶).

 Another clinical study demonstrated that saffron aqueous extract (15 mg twice daily) andcrocin (15 mg twice daily) were well tolerated by patients with schizophrenia during the study and no serious side effects were observed (Mousavi et al., 2015 ▶). 

The effectiveness of *C. sativus* as a treatment for depression in human studies was summarized in Table 2. 

**Anti-Parkinson effects**

Saffron and its components (mainly crocin, crocetin, and safranal) have been used in animal models with neurodegenerative diseases (Ochiai et al., 2007 ▶; Purushothuman et al., 2013 ▶). Crocin and safranal have inhibitory effect on ﬁbrillation of apo alpha-lactalbumin (a-alpha-LA), under amyloidogenic conditions which crocin was found to be more effective than safranal. Formation of toxic amyloid structures is related with various neurodegenerative diseases such as Alzheimer’s and Parkinson’s diseases (Ebrahim-Habibi et al. 2010 ▶).

Neuroprotective effects of seven-day administration of crocetin (25, 50 and 75µg/kg body weight, i.p.) against 6-hydroxydopamine (6-OHDA, 10 µgintrastriatal)-induced Parkinson's disease in rats have been reported. Reduction in dopamine utilization by tissues was suggested as a possible mechanism (Ahmad et al., 2005 ▶). In another study, the protective effect of saffron pre-treatment on dopaminergic cells in the substantia nigra pars compacta (SNc) and retina in a mouse model of acute MPTP (1-methyl-4-phenyl-1,2,3,6-tetrahydropyridine)-induced Parkinson's disease was examined. BALB/c mice received MPTP or saline over a 30-hour period. Animals in the saffron-treated group received Saffron (0.01% w/v) dissolved in the drinking water for five days and control groups received normal tap water. After the six days, the brains were processed for tyrosine hydroxylase (TH) immunochemistry and TH+ cells count was reported using the optical fractionator method. In both the SNc and retina, the MPTP-injected mice had a reduced number of TH+ cells (30-35%) compared to saline-injected controls. Pre- treatment of MPTP-injected mice by saffron increased both SNc and retinal TH+ cell counts (25-35%) and closed them to the control levels. It was concluded that saffron pre-treatment saved many dopaminergic cells in the SNc and retina from Parkinsonian (MPTP) insult in mice (Purushothuman et al., 2013 ▶).

**Effects of** ***C. sativus*** **on oxidative damages and neurotoxicity**

It has been reported that crocin 10 μM inhibited the formation of peroxidized lipids in cultured PC12 cells, moderately restored superoxide dismutase (SOD) activity and maintained neurons morphology. While the antioxidant effect of crocin was comparable to that of α –tocopherol, it was even more pronounced at some concentrations.

Administration of *C. sativus* stigma extract (100 mg/kg, p.o.) for 7 days before induction of cerebral ischemia by middle cerebral artery occlusion (MCAO) remarkably reduced SOD, catalase and Na, K-ATPase activities and glutamate and aspartate concentrations induced by ischemia in rats (Saleem et al. 2006 ▶). Treatment with saffron extract (5 and 25 mg/ml) and crocin (10 and 50 μM) could decrease the neurotoxic effect of glucose in PC12 cells. The results showed that glucose (13.5 and 27 mg/ml) reduced PC12 cells viability while cell death was reduced by saffron and crocin pretreatment (Mousavi et al., 2010 ▶). Another study showed that administration of saffron extract (200 mg/kg) and honey syrup (500 mg/kg) for 45 days reduced the aluminium chloride-induced neurotoxicity in mice (Shati et al. 2011 ▶). Other studies showed that safranal has some protective effects on different markers of oxidative damage in hippocampal tissue from ischemic rats (Hosseinzadeh and Sadeghnia, 2005 ▶) and in hippocampal tissue following quinolinic acid (QA) administration (Sadeghnia et al., 2013 ▶). Safranal also reduced extracellular concentrations of glutamate and aspartate (excitatory amino acids) in the hippocampus of anaesthetized rats following kainic acid administration (Hosseinzadeh et al. 2008b ▶).

In addition, crocin increased the activity of SOD and glutathione peroxidase (GPx) and remarkably reduced malondialdehyde (MDA) content in the ischemic cortex in rat model of ischemic stroke (Vakili et al., 2013 ▶). Co-administration of saffron extract with aluminium reversed aluminium-induced changes in monoamine oxidase (MAO-A, MAO-B) activity and the levels of lipid peroxidation in whole brain and cerebellum (Linardaki et al., 2013 ▶). 

It has been suggested that exposure to high levels of glucocorticoids or chronic stress may lead to oxidative injury in the hippocampus, which may impair learning and memory functions (Behl et al., 1997 ▶; McIntosh et al., 1998a). Saffron extract and crocin can improve learning and memory (Abe and Saito, 2000 ▶, Pitsikas et al., 2007 ▶). It was demonstrated that saffron and crocin can prevent oxidative stress in the hippocampus and avoid deficits in spatial learning and memory (Ghadrdoost et al., 2011 ▶). It has been reported that crocetin increases the antioxidant potential in brain and helps to fight against 6-OHDA-induced neurotoxicity (Ahmad et al., 2005 ▶).

The aqueous extract of saffron (50, 100 and 200 mg/kg) prevented diazinon (20 mg/kg)-induced increase of inflammation, oxidative stress and neuronal damage biomarkers (Moallem et al., 2014 ▶).

**Table2:** The effectiveness of *C. sativus* as a treatment for depression in human studies.

**Number of patients**	**Treatments**	**Time ofTreatment (weeks)**	**Results**	**References**
**30**	Stigma of *C. sativus*30 mg/day	6	The effect of stigma of *C. sativus*similar to imipramine in the treatment of mild to moderate depression	Akhondzadeh et al.,2004
**40**	Stigma of *C. sativus*30 mg/day	6	The outcome on the Hamilton depression rating scale Stigma of *C. sativus*could produce a significantly better than the placebo	Akhondzadeh et al.,2005
**40**	Stigma of *C. sativus*30 mg/day	6	The effect of stigma of *C. sativus*similar to fluoxetine in the treatment of mild to moderate depression	Noorbala et al.,2005
**40**	Petal of *C. sativus*30 mg/day	6	The outcome on the Hamilton depression rating scale Petal of *C. sativus*could produce a significantly better than the placebo	Moshiri et al.,2006
**40**	Petal of *C. sativus*15 mg bid (morning and evening)	8	Petal of *C. sativus*was found to be effective similar to fluoxetine in The treatment of mild to moderate depression	AkhondzadehBasti et al., 2007
**60**	*C. sativus*40 and 80 mg/day+ fluoxetine (30 mg)	6	Was effective to treatment of mild to moderate depressive disorders	Moosavi et al., 2014
**40**	Saffron(30 mg/day	6	Was effective as ﬂuoxetine (40 mg/day) in improving depressive symptoms of patients who are suffering from major depressive disorder (MDD)	Shahmansouri et al., 2014

**Effects of** ***C. sativus*** **on neuronal injury and apoptosis**

Crocin (30, 60 and 120 mg/kg) showed protective effect against ischemia/reperfusion injury and cerebral edema in a rat model of stroke and decreased infarct volume. Administration of crocin (60 mg/kg), one hour before, or one hour after the induction of ischemia, reduced brain edema (Vakili et al., 2013 ▶). 

The neuroprotective effects of crocetin in the brain injury in animal studies have been suggested to be related to its ability to inhibit apoptosis at early stages of the injury and its ability to promote angiogenesis at the subacute stage as directed by higher expression levels of vascular endothelial growth factor receptor-2 (VEGFR-2) and serum response factor (SRF) (Bie et al., 2011 ▶).

A recent study showed that crocin (50 mg/kg) prevented retinalganglion cells (RGCs) apoptosis after retinal ischemia/reperfusion injury via phosphatidylinositol 3-kinase/AKT (PI3K/AKT) signaling pathway. In addition, crocin increased Bcl-2/BAX ratio (Qi et al., 2013 ▶). Crocin (10 µM)could suppress tumor necrosis factor alpha (TNF-α)-induced expression of proapoptotic mRNA which releases cytochrome c from mitochondria and it was suggested that crocin inhibits neuronal cell death induced by both internal and external apoptotic stimuli (Soeda et al., 2001 ▶). Moreover, crocetin can inhibit H_2_O_2_-induced RGC-5 cell death and inhibit caspase-3 and caspase-9 activity (Yamauchi et al., 2011 ▶). 

In serum/glucose-deprived cells, lipid peroxidation may increase which can be inhibited by crocin. Crocin can suppress the activation of caspase-8 was and its antioxidant properties are more prounounced than α-tocopherol at the same concentration (Ochiai et al., 2004 ▶). In addition, crocin suppressed the activation of caspase-8 caused by serum/glucose deprivation (Ochiai et al., 2004a ▶).

Crocin and tricrocin remarkably suppressed membrane lipid peroxidation, caspase-3 activation and cell death in serum-deprived and hypoxic PC12 cells which were more marked than those of tricrocin. Crocetin has been suggested to have some linked glucose esters (Ochiai et al., 2007 ▶). The results of this study suggested that dicrocin and picrocrocin had no effect on cell survival (Ochiai et al., 2007 ▶). 

**Effects of** ***C. sativus*** **on neuroinflammation**

Crocin inhibited syncytin-1 and nitric oxide (NO)-induced astrocyte and oligodendrocyte cytotoxicity (Christensen, 2005 ▶) and reduced neuropathology in experimental autoimmune encephalomyelitis (EAE) with signiﬁcantly less neurological impairments. Syncytin-1 has been contributed to oligodendrocyte death and neuroinﬂammation (Christensen, 2005 ▶; Antony et al., 2004 ▶). Syncytin-1 is highly expressed in astrocytes, microglia and in the glial cells of multiple sclerosis lesions (Barnett and Prineas, 2004 ▶). 

Endoplasmic reticulum (ER) stress has been shown to be closely related to inflammatory pathways (Mori, 2009). It was shown that EAE increases the transcript levels of the ER stress genes *XBP-1/s* (Marciniak et al., 2004 ▶). Administration of crocin on day 7 post-EAE induction, suppressed ER stress and inflammatory gene expression in the spinal cord and also reduced the expression of ER stress genes *XBP-1/s* (Deslauriers et al., 2011 ▶).

***C. sativus*** **and the brain neurotransmitters**

Ettehadi et al. (2013) ▶ showed that the aqueous extract of saffron (50, 100, 150 and 250 mg/kg, i.p.) increased brain dopamine concentration in a dose-dependent manner. Moreover, the extract had no effect on brain serotonin or norepinephrine concentration. In addition, the results showed that the aqueous extract of saffron especially at the dose of 250 mg/kg triggered and increased the production of important neurotransmitters including dopamine and glutamate in rat brain (Ettehadi et al., 2013 ▶). 

The effects of saffron on conditioning place preference (CPP) induced by morphine has been reported to be similar to the effect of N-methyl-D-aspartate (NMDA) receptor antagonists (Hosseinzadeh et al., 2012 Lechtenberg et al., 2008 ▶). Furthermore, the analgesic eﬀect of saﬀron can be reduced by NMDA receptor antagonists (Nasri et al., 2011 ▶). Therefore an interaction with glutamatergic system for saffron of its components might be postulated. 

The NMDA receptors have also been well known to be involved in post-training memory processing by the amygdala and hippocampus (Izquierdo et al., 1992 ▶). The role of these receptors in morphine state-dependent learning has also been suggested (Zarrindast et al., 2006 ▶; Cestari and Castellano, 1997 ▶). Involvement of NMDA receptors in the eﬀects of *C. sativus* or its constituents on memory has been shown (Lechtenberg et al., 2008 ▶; Abe et al., 1999 ▶). The beneﬁcial eﬀects of saﬀron on memory have also been suggested to be mediated by the cholinergic system (Pitsikas and Sakellaridis, 2006 ▶; Ghadami and Pourmotabbed, 2009 ▶). 

***C. sativus*** **and opioids system**

Saﬀron aqueous (80–320 mg/kg) and ethanolic (400–800 mg/kg) extracts reduced morphine withdrawal signs induced by naloxone in mice (Hosseinzadeh and Jahanian, 2010 ▶). Also, crocin (200 and 600 mg/Kg) could reduce withdrawal sign without reducing locomotor activities (Amin and Hosseinzadeh, 2012 ▶; Hosseinzadeh and Jahanian, 2010 ▶). 

Intraperitoneal administration of ethanolic extract of saffron (10, 50 and 100 mg/Kg) and safranal (1, 5 and 10 mg/Kg) reduced theacquisition and expression of morphine CPP (Ghoshooniet al., 2011 ▶). Administration of crocin (400 and 600 mg/kg,i.p.) 30 min before morphine administration decreased the acquisition and reinstatement of morphine-induced CPP in mice (Imenshahidi et al., 2011 ▶). It has also been reported that 5 min after morphine (10 mg/kg) administration, injection of ethanolic extract of *C. sativus* stigma (5 and 10 µg/rat) into the nucleus accumbens shell part of rats, led to decrease in the time spent in drug paired side. In addition, injection of extract to the animals that received morphine (10 mg/kg), decreased the expression of morphine *(CPP)* (Mojabi et al., 2008). Injection of aqueous extract of saffron stigma (50, 100, 150 and 250mg/Kg,i.p.) showed an increased release of dopamine in rat brains. Also, this extract (only at 250 mg/Kg) significantly increased the release of glutamate (Ettehadi et al., 2013 ▶).

Administration of saﬀron extract (150 and 450 mg/kg) before retention trials also increased the time latency. So, saffron extract reduced morphine-induced memory impairment (Naghibi et al., 2012 ▶). Protective eﬀect of saﬀron extract against morphine-induced inhibition of spatial learning and memory in rat has also been suggested (Haghighizad et al., 2008 ▶). 

The effects of *C. sativus* on opioid receptors were showed in Table 3. 

**Table 3:** The effects of *C. sativus* on opioid system

***C. Sativus*** ** or its constituents**	**Dose**	**Results**	**References**
**Sa** **ﬀ** **ron**	150 and 450 mg/kg	Improved learning and memory impairment induced by morphine	Naghibi et al., 2012
**Sa** **ﬀ** **ron**	Aqueous (80, 160, 320 mg/kg) and ethanolic (400 and 800 mg/kg) extract	Reduced naloxone precipitated jumping	Ghoshooni et al., 2011; shams et al., 2009
**Crocin**	200 and 600 mg/kg	Reduced withdrawal sign without reducing locomotor activity	Amin and hosseinzadeh 2012
***C. sativus*** **stigma**	Alcohol extract (5 and 10 µg/rat)	Decrease in the time spent in drug paired side	Ghoshooni et al., 2011
**Crocin**	400 and 600 mg/kg	Decreased the acquisition and reinstatement of morphine-induced cpp	
**Saffron**	10, 50 and 100 mg/kg	Reduced the acquisition and expression of morphine cpp	
**Safranal**	1, 5 and 10 mg/kg	Reduced the acquisition and expression of morphine cpp	
**Saffron**	50, 100, 150 and 250mg/kg	Increased the release of dopamine in rat brains and increased the release of glutamate only in dose 250	Ettehadi et al., 2013

## Conclusion

Anti-oxidant and anti-inflammatory effects of the extracts of *C. sativus* and its constituents (crocetin, crocins, safranal) implies saffron therapeutic potential for various nervous system disorders. Based on the literature, beneficial effects of the plant and its components on neurodegenerative disorders such as Alzheimer and Parkinson's disease are mainly due to their interactions with cholinergic, dopaminergic and glutamatergic systems. It is assumed that saffron anticonvulsant and analgesic properties and its effects on morphine withdrawal and rewarding properties of morphine might be due to an interaction between saffron, GABA and opioid system.

According to human and animal studies, saffron and its constituents have been shown to be effective in the treatment of mild to moderate depression which may be because of an interaction with the serotonin and noradrenaline system. However, to have a detailed perspective of saffron effects on nervous system, more mechanistic investigations are highly advised.

## Conflict of interest

There is no conflict of interest.
